# Activation of Glucocorticoid Receptor Inhibits the Stem-Like Properties of Bladder Cancer via Inactivating the β-Catenin Pathway

**DOI:** 10.3389/fonc.2020.01332

**Published:** 2020-08-05

**Authors:** Congcong Xu, Mingwei Sun, Xiaozhen Zhang, Zhen Xu, Hiroshi Miyamoto, Yichun Zheng

**Affiliations:** ^1^Department of Urology, The Second Affiliated Hospital of Zhejiang University School of Medicine, Hangzhou, China; ^2^Department of Urology, The First Affiliated Hospital of Zhejiang University School of Medicine, Hangzhou, China; ^3^Department of Hepatobiliary and Pancreatic Surgery, The First Affiliated Hospital of Zhejiang University School of Medicine, Hangzhou, China; ^4^Department of Pathology and Laboratory Medicine, University of Rochester Medical Center, Rochester, NY, United States

**Keywords:** glucocorticoid receptor, bladder cancer, cancer stem cells, knockout, knockdown

## Abstract

**Background:** Glucocorticoid receptor (GR) signaling pathway has been shown to involve epithelial -to- mesenchymal transition which was implicated in the regulation of bladder cancer stem cells (CSCs) in our previous study. Herein, we aim to figure out how GR affects the stem-like properties of bladder cancer cells.

**Methods:** We used dexamethasone (DEX) treatment or gene-knockdown/-knockout techniques to activate or silence the GR pathway, respectively. Then we applied immunohistochemical staining and flow cytometry to assess the associations between the expression levels of GR and a stem cell surface marker CD44. Stem-like properties were assessed by reactive oxygen species (ROS), sphere-formation and side population assays. The expression levels of cancer stem cell-associated molecules were assessed by quantitative PCR and Western blotting. Tumor growth was compared using mouse xenograft models.

**Results:** In GR-positive bladder cancer cells, DEX significantly reduced the expression of CD44 as well as pluripotency transcription factors including β-catenin and its downstream target (C-MYC, Snail, and OCT-4), the rate of sphere formation, and the proportion of side populations, and induced the intracellular levels of ROS. By contrast, GR silencing in bladder cancer cells showed the opposite effects. In xenograft-bearing mice, GR silencing resulted in the enhancement of tumor growth.

**Conclusions:** These data suggested that GR activity was inversely associated with the stem-like properties of bladder cancer cells, potentially via inactivating the β-catenin pathway.

## Introduction

Bladder cancer (BCa), a heterogeneous disease with high metastasis and recurrence rates (1), ranks 13th in terms of deaths ranks (2) and has an estimated 81,190 new diagnoses and 17,240 deaths in the USA in 2018 (3). BCa can be divided into 2 categories consisting of non–muscle-invasive BCa (NMIBC) and muscle-invasive BCa (MIBC) (4). NMIBC accounts for around 70% of total BCa, and roughly twenty percent of them progress to MIBC. MIBC makes up ~30% of BCa with a high risk of death following distant metastases (5). Although modification of the disease management has improved the overall outcome including radical cystectomy, systemic chemotherapy, the 5-year relative survival rate is 76% (diagnosis years 1974–1985) (6) and 77% (diagnosis years 2008–2014) (7) in the America. The survival rate of patients with BCa has not obviously improved. Besides, Immunotherapy has gotten FDA approval in the preceding several years, both in the first-line or second-line for cisplatin-ineligible patients with BCa (8); but, response-rate ratio in the first line are about twenty-three percent (9, 10), and only fifteen percent in the second line (11). Hence in conclusion, new treatments and even ways to prevent tumor progression need to be developed.

Cancer stem cells (CSCs) introduced firstly in 1994, a distinct fraction of tumor cells, possess the ability to extensively self-renew and differentiate into progenitors (12). CSCs are considered responsible for cancer tumorigenicity, treatment resistance, relapse, and metastasis (13, 14). In 2009, chan et al. (15) isolated a subset of CD44(+)/CK5(+)/CK20(−) tumor-initiating cells in the BCa tissues for the first time and they found that CD44(+) BCa cells possessed differentiation potential. Thus, CD44 has always considered as a CSC marker.

Our group has long been involved in studying the effects of steroid hormone receptor signals in the progression of BCa from 2011 (16). We found that GR expression was downregulated in high-grade tumors (47%) compared with low-grade tumors (79%) (17). Additionally, we observed that dexamethasone (DEX) could induce mesenchymal-to-epithelial transition (MET) and inhibit invasion via activating the GR signaling pathway (18). Existing preclinical evidence indicated that glucocorticoid (GC) receptor (GR) had a function as a tumor suppressor in BCa (18, 19). We find that a lot of literature report epithelial-mesenchymal transition (EMT) will help to acquire CSCs properties in pancreatic cancer (20), breast cancer (21), and BCa (22).

Accordingly, we hypothesize that there may be a possible correlation between glucocorticoid receptor pathway and regulation of bladder CSCs, but remains unproven. Therefore, in this study, we want to explore the relationship between GR pathway and BCa stem cell-like properties.

## Materials and Methods

### Cell Culture

Human BCa cell lines (TCC-SUP), obtained from ATCC, were maintained in DMEM (GIBCO; CA; USA) with 10% FBS (Biological Industries; FL; USA) at 37°C under conditions of 5% CO_2_ and 20% O_2_. Cells were cultured in phenol-red free medium (GIBCO; CA; USA) containing 5% charcoal-stripped FBS (CS-FBS) (Biological Industries; FL; USA) at least 24 h before experimental treatment. DEX (Sigma; WI; USA) was used as GR agonist and mifepristone (Sigma; WI; USA) (RU486) was used as GR antagonist.

### Stable Cell Lines With GR Knockdown and GR Knockout

**Figure 1:**
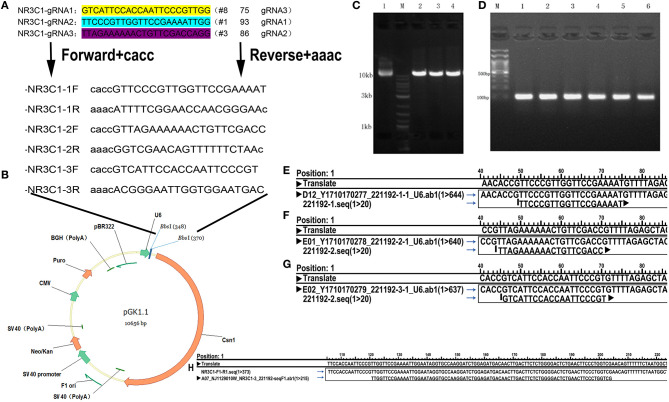
Schematic outline of the knockout process. **(A)** gRNA Target site selection: select the first exon of coding sequence and “CACC,” “AAAC” are added to the oligos. **(B)** Insertion positions and vector construction. **(C)** Electrophoresis of the plasmid after digestion. Lane 1, original plasmid (control); M, Markers; lanes 2–4, vector after cleavage. **(D)** Colony PCR product of selected clones. Clones 1–6 contain the desired 100 bp fragment, indicating that the target sequence oligonucleotides were correctly inserted. **(E–G)** Plasmid sequencing results showing alignment of the 1st **(E)** 2nd **(F)**, and 3rd **(G)** loci and confirming successfully construction of three knockout vectors. **(H)** Sequencing of a positive clone and alignment to the wild-type NR3C1 sequence showing deletion of 82bp, TTGGTTCCGAAAATTGGAATAGGTGCCAAGGATCTGGAGATGACAACTTGACTTCTCTGGGGACTCTGAACTTCCCTGGTCG.

Stable GR knockdown (KD) cell lines and their control lines were previously established (15). GR knockout (KO) cell lines were established by CRISPR/Cas9 genome editing mentioned by Pro. Zhang (23). We search GR genes (human NR3C1 gene; Gene ID: 2908) by using an online tool (www.ncbi.nlm.nih.gov/gene), and selected target sites within exon 1 by a web tool developed by Prof. Zhang (http://crispr.mit.edu/). The procedure was showed in Figure 1. GR-KO sequence shows deletion of 82bp comparing with the wild-type GR sequence (Figure 1H). KO cell lines were expended over 4 generations and were cultured ~3 weeks in order to acquire stable mutation.

### Immunohistochemical Staining

MIBC samples (*n* = 28), NMIBC samples (*n* = 21) and benign samples (*n* = 18) sourced from as the Second Affiliated Hospital of Zhejiang University School of Medicine approved by the ethics committee. Immunohistochemistry was performed as described previously (16). After dewaxing and hydrating tissue sections, antigen retrieval was performed by high pressure. Next, sections were incubated with 3% H_2_O_2_ to block endogenous peroxidase. Then sections were incubated with normal goat serum for 30 min at room temperature. The sections were incubated with primary antibody CD44 (dilution 1:200, #37259, CST) and GR (dilution 1:100, #3660, CST) at 4°C overnight. Then sections were incubated with the secondary antibody. Finally, after staining with DAB and hematoxylin, sections were dehydrated by alcohol and visualized under a microscope. All the stains were manually scored by one pathologist blinded to patient identity. Six random views were selected and one hundred cells within field were counted for analysis. The immunoreactive score was obtained by multiplying the percentage of positive cells (0–10% = negative or marginal; 11–40% = weak; 41–70% = moderate; 71–100% = strong).

### Reactive Oxygen Species Analysis

Cells (3 × 10^3^/96-well plates) were seeded in medium supplemented with CS-FBS for 24 h in the presence of DEX and/or RU486. Cells were stained with 10 μM DCFH-DA (Beyotime, SH, CHN) in phenol-red free DMEM without serum at 37°C for 20 min, washed with medium, and subjected to fluorescence plate reader (Thermo, NY, USA) (details in Supplementary Table 1).

### Sphere-Formation Assay

Cells (1 × 10^6^/6-well plates) were cultured in phenol-red free DMEM medium containing 5% CS-FBS supplemented with ligands (DEX/RU486) at 37°C in a CO_2_ incubator for 24 h. Then after 24 h, the cells were seeded in ultra-low-attachment 6-well plates (Corning Inc.) at a density of 2 × 10^4^ cells/well and maintained in a phenol-red free DMEM/f12 (Gibco; CA; USA) supplemented with insulin epidermal growth factor (20 ng/ml; Invitrogen), basic fibroblast growth factor (10 ng/ml; Invitrogen), N2 (Invitrogen; CA; USA) and B27 (Invitrogen; CA; USA). Spheres were imaged and counted under a phase-contrast microscope (Leica; BS; DEU) after 1 week (details in Supplementary Table 2).

### Side Population Analysis

Cells were seed at the 6-well plates supplemented with phenol-red free DMEM medium containing 5% CS-FBS 48 h before side population (SP) sorting. SP assays were carried out as described previously (24). In brief, cells were cultured with or without verapamil (150 μM; Sigma; WI; USA) at 37°C for 30 min and then stained with Hoechst 33,342 (5 μg/ml; Sigma; WI; USA) for 90 min. Finally, cells were counterstained with propidium iodide (2 μg/ml; Sigma; WI; USA) in order to discriminate dead cells. Stained cells were analyzed using a FACS Aria cell sorter (BD Biosciences; NJ; USA).

### Fluorescence-Activated Cell Sorting

Cells were harvested by non-EDTA trypsin and washed twice with PBS. Then cells were incubated CD44 antibody (Abcam; EPR18668) at 4°C for 30 min in the dark. After incubation, cells were washed third with ice cold PBS. The stained cells were sorted and analyzed using a BD FACS Canto™ II (BD Biosciences). The percentage of CD44+ sorted cells were evaluated with the FACS Canto™ cell sorter (details in Supplementary Table 3).

### Western Blotting

Western blotting protocol were carried out as described previously (16). The cell culture dish was placed on ice and washed with ice-cold PBS. Then cells were lysed by RIPA containing 1x protease inhibitor cocktail for 1 h on ice. Next, centrifugated at 15,000 g for 15 min at 4°C, and collected the supernatants. Total protein (35 μg/15 μl) as measured by the bicinchoninic acid protein assay was separated by electrophoresis on SDS-PAGE gels, and transferred onto polyvinylidene fluoride membrane to incubated with anti-CD44 antibody (ab51037, Abcam), anti-GR antibody (ab228972, Abcam), anti-β-catenin antibody (ad224803, Abcam), anti-C-MYC antibody (#5605, CST), anti-Snail antibody (#3879, CST), anti-OCT-4 antibody (ab109183, Abcam), anti-GAPDH antibody (#5174, CST), overnight at 4°C. Finally, the membrane was incubated at 4°C with the appropriate secondary antibodies, and autoradiography was performed with the ChemiDoc MP Imaging System (Bio-Rad).

### Reverse Transcription and Real-Time PCR

Total RNA was extracted from cultured cells with TRIzolZ (Sigma). RNA was reverse transcribed to generate cDNA by using First Strand cDNA Synthesis Kit (Invirtrogen). cDNA was then subjected to real-time PCR by SYBR Green (Takara), as described previously (25). The next primer pairs were used for PCR: GR (forward: 5′-CTGTCGCTTCTCAATCAGACTC-3′; reverse: 5′- CCCAGGTCATTTCCCATCACTT-3′), β-catenin (forward: 5′-TGCAGTTCG CCTTCACTATG-3′; reverse: 5′-ACTAGTCGTGGAATG GCACC-3′), C-MYC (forward: 5′-GCCCAGTGAG GATATCTGGA-3′; reverse: 5′-ATCGCAGATGAAGC TCTGGT-3′), GAPDH (forward: 5′-CGCTCTC TGCTCCTCCTGTTC-3′, reverse: 5′-ATCCGTTGACTC CGACCTTCAC-3′) (details in Supplementary Table 4).

### *In vivo* Experiments

Seven-week-old male severe combined immunodeficient (SCID) mouse were randomly divided into 3 groups and each mouse were transplanted with TCCSUP GR-positive cells, GR-KD cells or GR-KO cells. Growth of the tumor transplanted subcutaneously were determined weekly by vernier caliper measurement. When the sizes of all tumors in each group reached 20 mm^3^, slow-releasing pellets [dexamethasone (0.5 mg/mouse) or placebo, Innovative Research of America] were injected with a precision trocar. After 5 weeks of treatment, the mice were killed, then tumor volume was estimated as follows: tumor volume = (short axis)^2^ × (long axis) × 0.5.

### Statistical Analyses

Each experiment was carried out at least three times. Continuous data were recorded as mean SD if normally distributed or mean rank if not normally distributed, and analyzed with Student's *t-*test if normally distributed or Wilcoxon rank sum test if non-normally. Categorical data were analyzed with Fisher's exact test or chi-square test. Date analyses were performed using SPSS 23.0 (SPSS, Chicago, IL, USA) and Prism software (GraphPad, CA, USA) for Windows. *p* < 0.05 was considered statistically significant.

## Results

### Down-Regulation Expression of GR in Knockdown and Knockout Cell Lines

The KO positive clones were sequenced, and the result revealed a deletion of 82 bp compared with the wild-type GR sequence (Figure 1H). Next, we examined the expression of GR in TCC-SUP control, KD, and KO cell lines by QPCR and western blot analysis. Silencing of GR expression in KD and KO lines was then confirmed.

### CD44 Is Up-Regulated, but GR Is Down-Regulated in BCa

**Figure 2:**
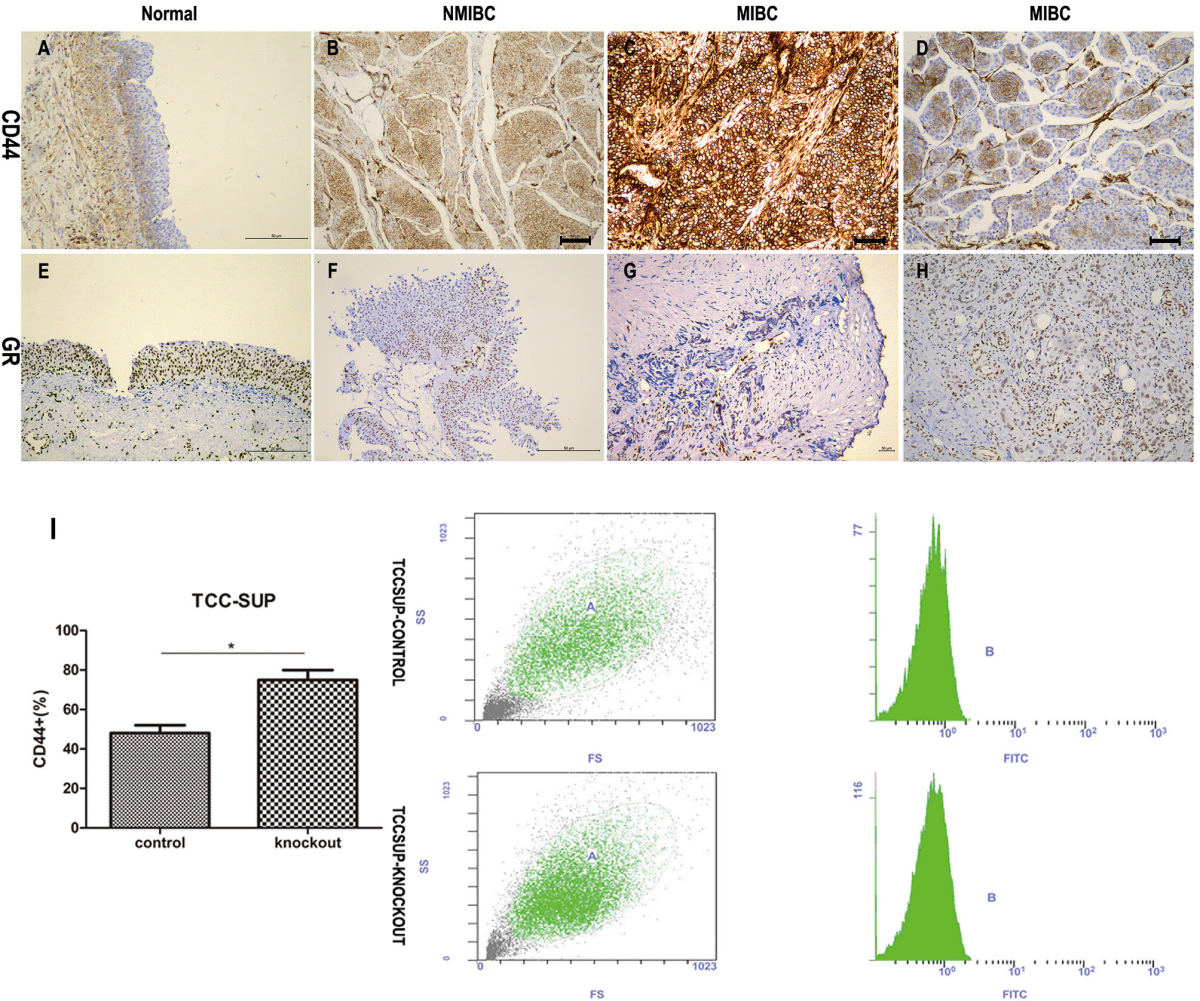
The relationship between CD44 and GR. **(A–H)** The immunochemical expression of CD44 **(A–D)** and GR **(E–H)**: CD44 antigen is a cell-surface glycoprotein; GR is expressed in the cytosol and nuclei of cells; **(A)** CD44 negative staining in non-neoplastic urothelium; **(B)** CD44 moderate staining in NMIBC; **(C)** CD44 strong staining in MIBC; **(D)** CD44 weak staining in MIBC; **(E)** GR strong staining in non-neoplastic urothelium; **(F)** GR weak staining in NMIBC; **(G)** GR negative staining in MIBC; **(H)** GR moderate staining in MIBC. **(I)** GR-control/GR-knockout cells cultured with 10% FBS for 24 h were used for flow cytometry analysis. Each value for CD44 positive rate represents the mean (+SD) from 3 independent experiments. *p < 0.05.

All parameters of immunohistochemical detection are summarized in Table 1 and Figures 2A–H. For CD44 staining, 26 (92.9%; 4 weak, 8 moderate, 14 strong) of 28 MIBC tissues; 18 (85.7%; 7 weak, 9 moderate, 2 strong) of 21 NMIBC specimens; 11 (61.1%; 9 weak, 2 moderate) of 18 benign bladder tissue were positive. We could find there were statistically significant differences between the tumor (MIBC + NMIBC) group and non-neoplastic urothelium group (*p* < 0.001), and between MIBC group and NMIBC group (*p* = 0.017). In addition, the rate of CD44 positivity was significantly higher in tumor than in non-neoplastic tissues (*p* = 0.012). For GR staining, 21 (75%; 15 weak, 6 moderate) of 28 MIBC, 19 (90.5%, 7 weak, 10 moderate, 2 strong) of 21 NMIBC, and 18 (100%, 6 weak, 9 moderate, 3 strong) of 18 non-neoplastic urothelial tissues were positive. The results proved that there were statistically significant differences between MIBC group and NMIBC group; tumor group and non-neoplastic urothelium group (*p* = 0.041, *p* = 0.046, respectively). Besides, the rate of GR positivity was significantly lower was in tumor than in non-neoplastic tissues (*p* = 0.048).

**Table 1:** The expression of CD44/GR in BCa tissues and non-neoplastic tissues.

	**Negative (CD44/GR)**	**Positive (+)**			***P-*****value**	
		**Weak (CD44/GR)**	**Moderate (CD44/GR)**	**Strong (CD44/GR)**	**MIBC vs. NMIBC**	**MIBC + NMIBC vs. Normal**
MIBC	2 (7.1%)/7 (25.0%)	4 (14.3%)/15 (53.6%)	8 (28.6%)/6 (21.4%)	14 (50%)/0 (0%)	CD44 (χ^2^) GR (χ^2^) CD44 (+) GR (+)	0.017^※^ < 0.001^※^ 0.041^※^ 0.046^※^ 0.693^※^ 0.012^☆^ 0.522^*^ 0.048^⋆^
NMIBC	3 (14.3%)/2 (9.5%)	7 (33.3%)/7 (33.3%)	9 (42.9%)/10 (47.6%)	2 (9.5%)/2 (9.5%)		
Non-neoplastic urothelium	7 (38.1%)/0 (0%)	9 (50.0%)/6 (33.3%)	2 (11.1%)/9 (50.0%)	0 (0%)/3 (16.7%)		

*CD44 (χ^2^) = Negative vs. Weak vs. Moderate vs. Strong*.

*GR (χ2) = Negative vs. Weak vs. Moderate vs. Strong*.

*CD44 (+) = The rate of CD44 positivity including weak, moderate, and strong; Negative vs. Weak + Moderate + Strong*.

*GR (+) = The rate of GR positivity including weak, moderate, and strong; Negative vs. Weak + Moderate + Strong*.

*Normal = Non-neoplastic urothelium*.

※ *Fisher's exact test*.

☆ *Chi square test*.

* *Chi square test for continuity correction*.

In addition, we performed flow cytometry (Figure 2I), and found that GR-KO cell lines had more CD44 positive cells than GR-positive cell lines (74.96% ± 0.071 for GR-KO cell vs. 48.02% ± 0.037 for GR-positive cell, *p* = 0.042).

### Dexamethasone Suppressed the Stem-Like Properties of BCa Cells, Knockdown, and Knockout of GR Enhances the Stem-Like Properties

**Figure 3:**
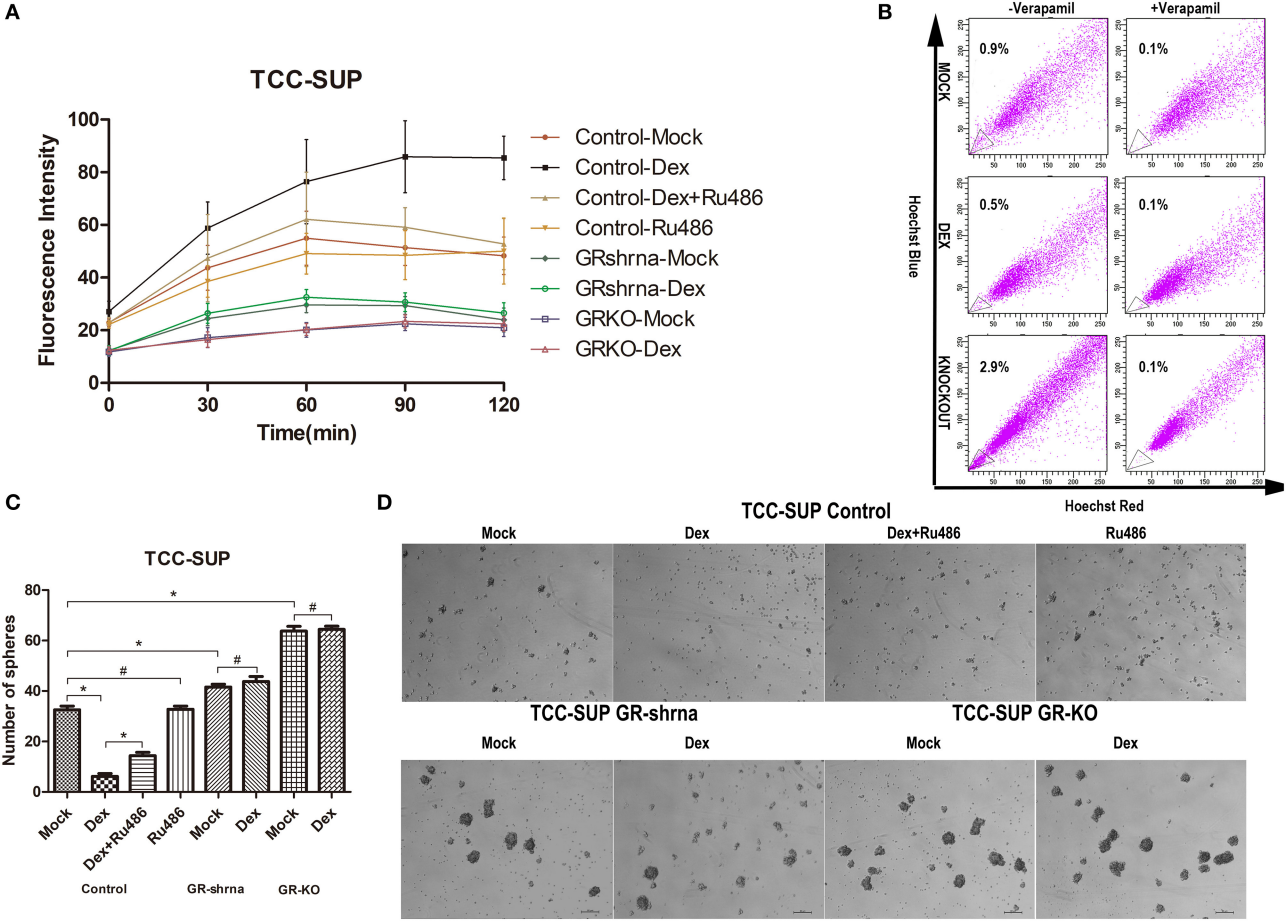
Inactivation of GR enhances the stem-like properties of BCa cells. **(A)** GR-Control/-Knockdown/-Knockout cells were cultured with ethanol (mock), 100 nmol/L dexamethasone, and 1 mmol/L RU486 for 24 h and subsequently treated with H_2_O_2_ (400 uM) to induce oxidative stress. ROS concentrations were determined by 2′,7′-dichlorofluorescein diacetate assay staining. **(B)** GR-Control/-Knockout cells were cultured with ethanol (mock) or 100 nmol/L dexamethasone for 48 h. The control group were under the verapamil pre-incubated. Side population cells were analyzed by fluorescence-activated cell sorting. **(C,D)** GR-Control/-Knockdown/-Knockout cells were incubated with DMEM/F12 supplemented with B27, N2, EGF, and bFGF in the presence of ethanol (mock), 100 nmol/L dexamethasone, and 1 mmol/L RU486. Cell clone was counted after 1 week. *P < 0.05. ^#^*P* > 0.05.

Cancer stem-cell contain lower levels of ROS than non-tumorigenic cells (26). Cells (3 × 10^3^) seeded in 96-well plates were incubated with medium supplemented with or without CS-FBS containing ligands (DEX/RU486). After 48 h of treatment, we compared ROS levels in cells. The levels of ROS were detected through DCFH-DA that could be oxidized to fluorescent DCF, so fluorescence intensity is proportionate to the levels of ROS. The results (Figure 3A) showed that (1) GR-positive + DEX group contained significantly higher concentrations of ROS than other groups in the 60th, 90th, 120th min; (2) GR-KD + Mock and GR-KO + Mock cell lines contained significantly lower concentrations of ROS than GR-positive + Mock cell lines in the 30th, 60th, 90th, 120th min; (3) GR-KO + Mock group contained significantly lower concentrations of ROS than GR-KD + Mock group in the 60th min.

Diverse cancer cell lines possessed side populations that having stem cell-like features including BCa (27). We evaluated the SP fraction in GR-positive cells, GR-positive cells supplemented with DEX, GR-KO cells (Figure 3B). 0.9, 0.5, 2.9% SP cells were observed in GR-positive cells, GR-positive cells supplemented with DEX, GR-KO cells, respectively. The SPs disappeared after treatment with the calcium channel blocker, verapamil, thus resulting in the inhibition of Hoechst33342 uptake.

Sphere formation has been well-described as a typical characteristic of CSCs that reflects the potential for self-renewal (14). We verified the function of GR in tumor formation by sphere formation assay (Figures 3C,D). DEX significantly suppressed sphere formation in GR-positive cells (6.2 ± 2.1 for DEX group vs. 32.6 ± 2.9 for mock group), but not in GR-KD and GR-KO cells. And this induction was abolished by RU486, and GR-KD and GR-KO cells exhibited a higher frequency of spherical colonies than GR-positive cells (41.6 ± 2.2 for KD and 63.8 ± 3.7 for KO), and GR-KO group was higher when compared to each other.

### Dexamethasone Decreased Cells Express Stem Cell Markers, Knockdown, and Knockout of GR Increased Cells Express Stem Cell Markers

**Figure 4:**
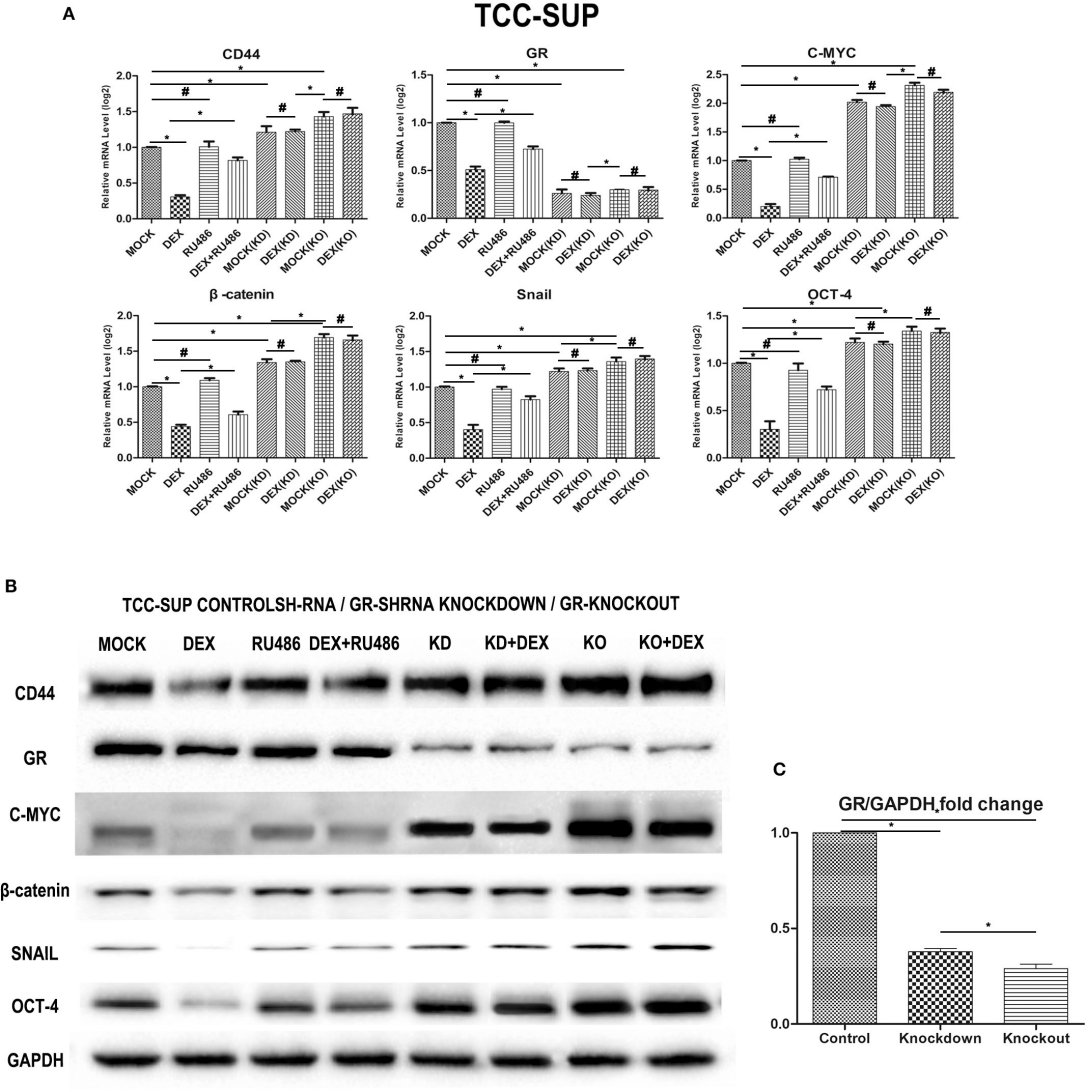
Dexamethasone decreased the expression of cancer stem cell markers. **(A)** GR-Control/Knockdown/Knockout treated with ethanol (mock), 100 nmol/L dexamethasone, and 1 mmol/L RU486 for 48 h were subjected to RNA extraction, RT and real-time PCR. Expression of each specific gene was normalized to that of GAPDH. Transcription amount is presented relative to that of mock treatment in each cell line. Each value represents the mean ± SD from at least 3 independent experiments. ^*^P < 0.05; ^#^*P* > 0.05. **(B)** GR-Control/Knockdown/Knockout cultured for 48 h in the presence of ethanol (mock), 100 nmol/L dexamethasone, and 1 mmol/L RU486 were analyzed on western blot analysis, using an antibody to CD44, GR, C-MYC, β-catenin, Snail, Oct-4. GAPDH was used as a reference control. Data were obtained from at least three independent experiments. The data are presented as mean ± SD. **(C)** GR bands of GR- Control/Knockdown/Knockout cell lines were analyzed by Image J. *P < 0.05.

We performed a human cancer stem cell marker PCR array. The QPCR analysis indicated that GC/GR decrease the expression of the target genes that play a key role in stem cell (Figure 4A). DEX decreased the levels of CD44, C-MYC, β-catenin, Snail, OCT-4 by 69.7, 78.7, 56.1, 59.8, 69.8% in GR-positive lines, respectively, and inhibitory effects of DEX on the expression of these 5 genes were antagonized by RU486. In GR-KD/KO line, the levels of CD44, C-MYC, β-catenin, Snail, OCT-4 increased by 21.3/43.1%, 2.0/31.4%, 34.1/66.0%, and 22.0/36.0%, 21.0/0.34.0%, respectively. Besides, the levels of these 5 genes in the GR-KO lines were significantly higher than in the KD lines, and inhibitory effects of DEX on the expression of these 5 genes were not significant. Consistent with the QPCR data, the expression of these proteins was weaker in the DEX group, and stronger in the KD/KO group, as demonstrated by western blotting (Figures 4B,C).

### GR-KO Cells Display Highly Tumorigenic Behavior *in vivo*

**Figure 5:**
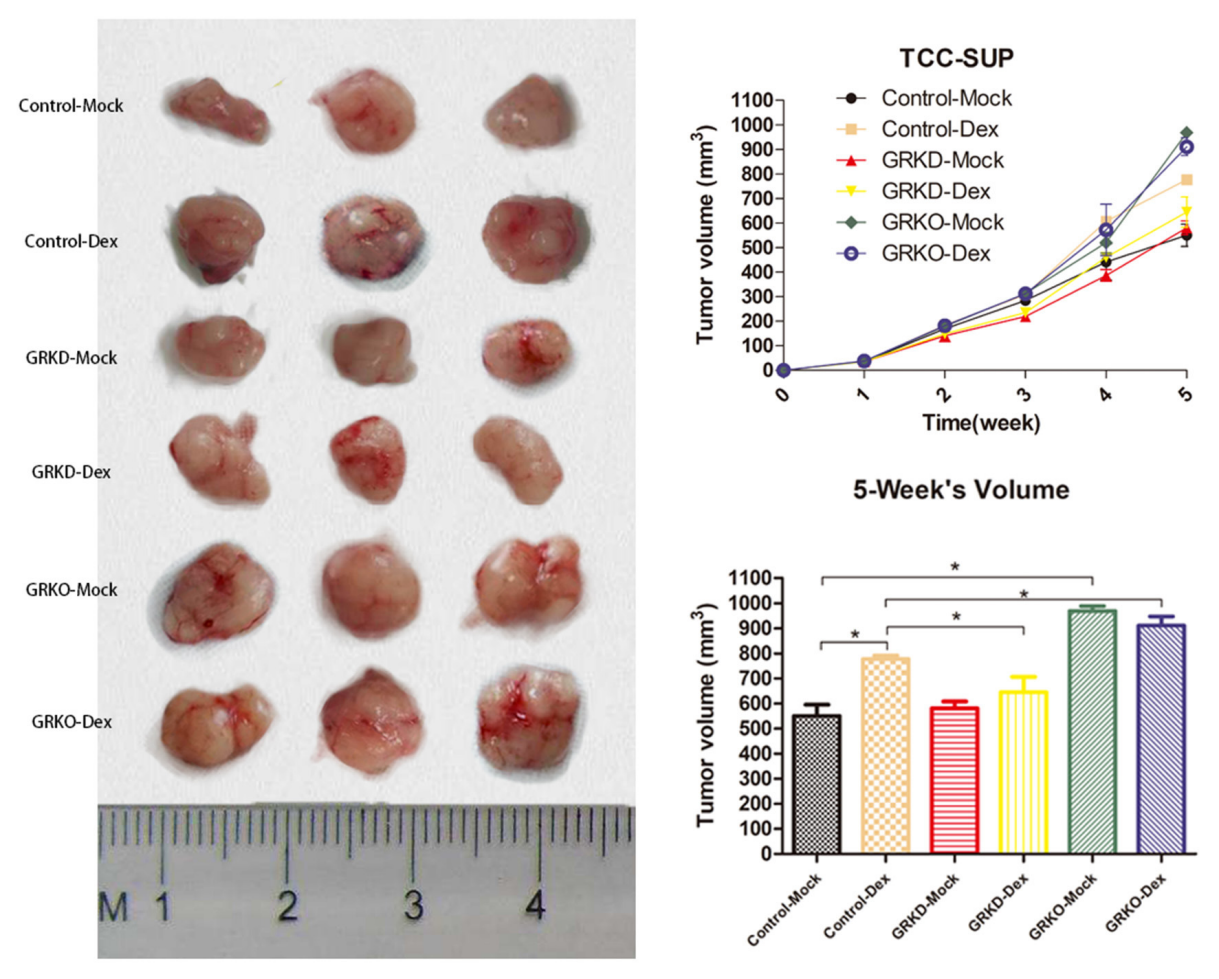
The subcutaneous tumorigenicity in nude mice. GR-control/-knockdown/-knockout cells were implanted subcutaneously into the nude mice, and treatment (injection of dexamethasone or placebo pellet) began when estimated tumor volume reached 20 mm^3^ (each group, *n* = 3). Tumor volume was monitored for 5 weeks (right top, mean ± SD), and tumors were then harvested and measured (right bottom, mean ± SD). *P < 0.05.

As tumorigenic capacity is a major characteristic of cancer stem cells, GR-positive cells, GR-KD cells or GR-KO cells were subcutaneously injected into SCID mice for transplanted tumorigenicity analysis (Figure 5). GR-KO tumors in placebo-mice were larger than GR-positive (76.1%) and GR-KD tumors (66.9%) after 5 weeks. In the GR-positive cells, the tumors of dexamethasone-treated group were larger than placebo group (41.2%). In the GR-KD or KO cell lines, there were not significantly difference between dexamethasone-treated group and placebo group. Besides, GR-positive and GR-KO tumors were larger than GR-KD (20.5/41.3%) in dexamethasone-treated mice.

## Discussion

Hormone therapy for BCa still remained doubt and dispute comparing to hormone therapy for prostate cancer. Since 2011, our group has long been involved in studying the effects of steroid hormone receptor signals in the progression of BCa. Firstly, we found GR activation resulted in inhibition of BCa cell invasion, but at the same time inhibition of apoptosis (18). Next, we tried to explore its comprehensive effects in the BCa patients, and found that the expression levels of GR were lower in bladder tumors compared with bladder non-neoplastic tissue and in high-grade/MI tumors compared with low-grade/NMI tumors (28). Finally, we compared the inhibitory effects of 11 GR ligands, and found that prednisone significantly suppressed their neoplastic transformation (29). Previous studies indicated that GR might be a tumor-suppressor gene in the BCa, but the internal cause has been unknown. Now, we try to validate the relationship between GR signal pathway and stem cell-like properties. In this study, we introduced CRISPR/Cas9 gene-knockout techniques into altering GR gene sequence that result in an inactivated gene compared to previous studies. KO has been shown to achieve more efficient gene silence and loss-of-function than KD.

CSCs are a kind of unique cancer cells, and these cells can stimulate tumor growth through self-renewal (30). The sphere-formation assay confirmed that DEX could decrease self-renewal capacity of BCa cells. In addition, our ROS analysis suggested that levels of ROS were higher in the DEX treatment group than GR-KD or -KO group. Phillips et al. (31) founded that the levels of ROS were lower in breast CSCs than in non-stem cancer cells, contributing to tumor radio-resistance.

CD44, relevant in treatment resistance, has been proven to be a cancer stem cell surface marker of BCa (32). Our immunohistochemical study in 67 cystectomy specimens illustrated the lower the level of GR expression, the higher the level of CD44 expression. Then we performed QPCR and western blotting to confirm that the activation of glucocorticoid receptor signal pathway decreased CD44 transcription and translation. The FACS assay also indicated that the expression level of CD44 rose when GR genes were knocked out *in vitro* experiment. Besides, previous studies (17, 18) have confirmed that weak positivity of GR significantly correlated with recurrence and progression of BCa, and strong glucocorticoid receptor had correlate with better prognosis. Therefore, these results might suggest that GR predicted low-risk of BCa patients, which could be associated with CSCs hypothesis.

OCT-4 and C-MYC were proven to be the major transcription factors that are capable of rewriting stemness, and were regarded as cancer stem cell markers (33, 34). Additionally, WNT/β-catenin signaling pathway is considered to play a critical role in CSC maintenance (35). Activation of WNT signaling induces β-catenin translocating into the nucleus to promote expression of many genes (e.g., Snail) involved in regulating CSCs (36, 37). According our results, we found that β-catenin signaling pathway was inhibited and its downstream functional genes (Snail) reduced when GR signaling pathway was activated. We could verify whether GR regulates CSCs though β-catenin pathway in the further analyses.

In the mouse xenograft models, firstly we found that GR-KO tumors in placebo-mice were larger BCa than GR-positive and GR-KD tumors, which indicated the loss of GR genes could enhance tumorigenicity in BCa. However, GR-positive tumors in DEX treatment-mice were larger than GR-positive tumors in placebo-mice. Because DEX itself could inhibited BCa apoptotic cells death by downregulation of cleaved caspase-3 expression (18). Finally, GR-KD tumors was smaller than GR-positive tumors in DEX treatment-mice. In our opinion, the cause of this phenomenon was because the effect of GR was not completely blocked in GR-KD cells, so on the one hand, GR-KD cells could not possess strong tumorigenicity like KO cells, on the other hand, it partly blocked the function of glucocorticoids on the apoptosis inhibition. Further analyses are required to elucidate an underlying mechanism of glucocorticoid receptor–mediated tumorigenicity.

As we all know, although GCs are not clinically used to suppress BCa growth, GCs are used for prevention of side effects of chemotherapy and to improve the quality of life of patients with BCa (38). Our research team has been working on the role of GR in the progression of BCa, and found that GCs could repress invasion and metastasis but promote proliferation (18). In the past we thought GCs could promote proliferation via inhibiting apoptosis. However, Takeishi et al. (39) put forward that CSCs-wake-up therapeutic strategy that promoted the entry of CSCs into the cell cycle would give rise to cancer cell proliferation after analyzing many relevant clinical trials in their article. Cancer stem cells are maintained in a non-proliferative state (G0 phase) and enter the cell cycle infrequently, whereas they could be woken up and then differentiate and proliferate (40). We have performed cell cycle analysis (18) and found GCs could enhance cell cycle at the G1 phase. In summary, we inferred that DEX might induce the entry of CSCs into the cell cycle, so promote proliferation.

Although CSCs have been isolated from numerous cancers and could explain the poor results of many anticancer therapies, few data provide direct evidences to prove that CSCs have relevance to clinical practice. However, given the fact that CSCs play a key role in cancer tumorigenicity, relapse, and metastasis, regulatory factors participating in CSC self- renewal may be promising targets to suppress tumor progression.

Although CSCs have been isolated from numerous cancers and could explain the poor results of many anticancer therapies, few data provide direct evidences to prove that CSCs have relevance to clinical practice. However, given the fact that CSCs play a key role in cancer tumorigenicity, relapse, and metastasis, regulatory factors participating in CSC self- renewal may be promising targets to suppress tumor progression. Of course, our study has several limitations. Firstly, underlying mechanisms of the regulation of CSCs by GR are not confirmed. In the following experiments, we will verify whether GR regulates the stem-like properties of BCa by WNT/β-catenin signal pathway. In addition, we want to validate the relationship between GR and radio-resistance when we find the lower level of ROS in the GR-KD and -KO group, but we cannot carry up irradiation treatment due to the lack of relevant radiation source. Finally, we apply xenograft mouse model to simulate tumor growth *in vivo*. However, GCs will suppress immune system, so SCID mice with a compromised immune system cannot simulate real BCa patients.

## Conclusion

On the basis of our current results, the expression levels of GR were found to be positively correlated with clinical stage and grade. Besides, DEX would have the opportunity to be applied in order to reduce recurrence and metastasis of BCa patients in clinic.

## Data Availability Statement

The raw data supporting the conclusions of this article will be made available by the authors, without undue reservation.

## Ethics Statement

The studies involving human participants were reviewed and approved by Human Ethics Review Committee of Zhejiang University Second Affiliated Hospital. The patients/participants provided their written informed consent to participate in this study. The animal study was reviewed and approved by Animal Ethics Committee of Zhejiang University Second Affiliated Hospital.

## Author Contributions

CX, HM, and YZ: conceptualization. CX, MS, and YZ: methodology. XZ and ZX: software. CX: validation, formal analysis, investigation, and writing—original draft preparation. YZ: resources, writing—review and editing, supervision, project administration, and funding acquisition. MS: data curation. CX and MS: visualization. All authors contributed to the article and approved the submitted version.

## Conflict of Interest

The authors declare that the research was conducted in the absence of any commercial or financial relationships that could be construed as a potential conflict of interest.

## References

[1] Burger M Catto JW Dalbagni G Grossman HB Herr H Karakiewicz P. Epidemiology and risk factors of urothelial bladder cancer. Eur Urol. (2013) 63:234–41. 10.1016/j.eururo.2012.07.033 22877502

[2] Antoni S Ferlay J Soerjomataram I Znaor A Jemal A Bray F. Bladder cancer incidence and mortality: a global overview and recent trends. Eur Urol. (2017) 71:96–108. 10.1016/j.eururo.2016.06.010 27370177

[3] Siegel RL Miller KD Jemal A Cancer statistics, 2018. CA Cancer J Clin. (2018) 68:7–30. 10.3322/caac.21442 29313949

[4] Flaig TW Spiess PE Agarwal N Bangs R Boorjian SA Buyyounouski MK. NCCN guidelines insights: bladder cancer, version 5.2018. J Natl Compr Canc Netw. (2018) 16:1041–53. 10.6004/jnccn.2018.0072 30181416

[5] Sun M Trinh QD. Diagnosis and staging of bladder cancer. Hematol Oncol Clin North Am. (2015) 29:205–18. 10.1016/j.hoc.2014.10.013 25836929

[6] Silverberg E Boring CC Squires TS. Cancer statistics, 1990. CA Cancer J Clin. (1990) 40:9–26. 10.3322/canjclin.40.1.9 2104569

[7] Siegel RL Miller KD Jemal A Cancer statistics, 2019. CA Cancer J Clin. (2019) 69:7–34. 10.3322/caac.21551 30620402

[8] Hsu MM Balar AV. PD-1/PD-L1 combinations in advanced urothelial cancer: rationale and current clinical trials. Clin Genitourin Cancer. (2019) 17:e618–26. 10.1016/j.clgc.2019.03.009 31005473

[9] Slater RM Hughes NC. A simplified method of treating burns of the hands. Br J Plast Surg. (1971) 24:296–300. 10.1016/S0007-1226(71)80074-7 5568632

[10] Zhou TC Sankin AI Porcelli SA Perlin DS Schoenberg MP Zang X. A review of the PD-1/PD-L1 checkpoint in bladder cancer: from mediator of immune escape to target for treatment. Urol Oncol. (2017) 35:14–20. 10.1016/j.urolonc.2016.10.004 27816403

[11] Sonpavde G Sternberg CN Rosenberg JE Hahn NM Galsky MD Vogelzang NJ. Second-line systemic therapy and emerging drugs for metastatic transitional-cell carcinoma of the urothelium. Lancet Oncol. (2010) 11:861–70. 10.1016/S1470-2045(10)70086-3 20537950

[12] Lapidot T Sirard C Vormoor J Murdoch B Hoang T Caceres-Cortes J. A cell initiating human acute myeloid leukaemia after transplantation into SCID mice. Nature. (1994) 367:645–8. 10.1038/367645a0 7509044

[13] Clarke MF Fuller M. Stem cells and cancer: two faces of eve. Cell. (2006) 124:1111–5. 10.1016/j.cell.2006.03.011 16564000

[14] Dalerba P Cho RW Clarke MF. Cancer stem cells: models and concepts. Annu Rev Med. (2007) 58:267–84. 10.1146/annurev.med.58.062105.204854 17002552

[15] Chan KS Espinosa I Chao M Wong D Ailles L Diehn M. Identification, molecular characterization, clinical prognosis, and therapeutic targeting of human bladder tumor-initiating cells. Proc Natl Acad Sci USA. (2009) 106:14016–21. 10.1073/pnas.0906549106 19666525 PMC2720852

[16] Zheng Y Izumi K Yao JL Miyamoto H. Dihydrotestosterone upregulates the expression of epidermal growth factor receptor and ERBB2 in androgen receptor-positive bladder cancer cells. Endocr Relat Cancer. (2011) 18:451–64. 10.1530/ERC-11-0010 21613411

[17] Ishiguro H Kawahara T Zheng Y Netto GJ Miyamoto H. Reduced glucocorticoid receptor expression predicts bladder tumor recurrence and progression. Am J Clin Pathol. (2014) 142:157–64. 10.1309/AJCPU8UCEZYG4WTV 25015855 PMC4337856

[18] Zheng Y Izumi K Li Y Ishiguro H Miyamoto H. Contrary regulation of bladder cancer cell proliferation and invasion by dexamethasone-mediated glucocorticoid receptor signals. Mol Cancer Ther. (2012) 11:2621–32. 10.1158/1535-7163.MCT-12-0621 23033490

[19] Zheng Y Ishiguro H Ide H Inoue S Kashiwagi E Kawahara T. Compound A inhibits bladder cancer growth predominantly via glucocorticoid receptor transrepression. Mol Endocrinol. (2015) 29:1486–97. 10.1210/me.2015-1128 26322830 PMC5414678

[20] Luo Z Li Y Zuo M Liu C Tian W Yan D. Effect of NR5A2 inhibition on pancreatic cancer stem cell (CSC) properties and epithelial-mesenchymal transition (EMT) markers. Mol Carcinog. (2017) 56:1438–48. 10.1002/mc.22604 27996162 PMC5392129

[21] Mani SA Guo W Liao MJ Eaton EN Ayyanan A Zhou AY. The epithelial-mesenchymal transition generates cells with properties of stem cells. Cell. (2008) 133:704–15. 10.1016/j.cell.2008.03.027 18485877 PMC2728032

[22] Garg M. Urothelial cancer stem cells and epithelial plasticity: current concepts and therapeutic implications in bladder cancer. Cancer Metastasis Rev. (2015) 34:691–701. 10.1007/s10555-015-9589-6 26328525

[23] Huang P Tong D Sun J Li Q Zhang F. Generation and characterization of a human oral squamous carcinoma cell line SCC-9 with CRISPR/Cas9-mediated deletion of the p75 neurotrophin receptor. Arch Oral Biol. (2017) 82:223–32. 10.1016/j.archoralbio.2017.06.004 28654784

[24] Wang J Guo LP Chen LZ Zeng YX Lu SH. Identification of cancer stem cell-like side population cells in human nasopharyngeal carcinoma cell line. Cancer Res. (2007) 67:3716–24. 10.1158/0008-5472.CAN-06-4343 17440084

[25] Ishiguro H Kawahara T Zheng Y Kashiwagi E Li Y Miyamoto H Differential regulation of bladder cancer growth by various glucocorticoids: corticosterone and prednisone inhibit cell invasion without promoting cell proliferation or reducing cisplatin cytotoxicity. Cancer Chemother Pharmacol. (2014) 74:249–55. 10.1007/s00280-014-2496-7 24880571

[26] Diehn M Cho RW Lobo NA Kalisky T Dorie MJ Kulp AN. Association of reactive oxygen species levels and radioresistance in cancer stem cells. Nature. (2009) 458:780–3. 10.1038/nature07733 19194462 PMC2778612

[27] Oates JE Grey BR Addla SK Samuel JD Hart CA Ramani VA. Hoechst 33342 side population identification is a conserved and unified mechanism in urological cancers. Stem Cells Dev. (2009) 18:1515–22. 10.1089/scd.2008.0302 19260804

[28] Ide H Miyamoto H. Steroid hormone receptor signals as prognosticators for urothelial tumor. Dis Markers. (2015) 2015:840640. 10.1155/2015/840640 26770009 PMC4685115

[29] Ide H Inoue S Mizushima T Kashiwagi E Zheng Y Miyamoto H. Role of glucocorticoid signaling in urothelial tumorigenesis: inhibition by prednisone presumably through inducing glucocorticoid receptor transrepression. Mol Carcinog. (2019) 58:2297–305. 10.1002/mc.23118 31535408

[30] Nguyen LV Vanner R Dirks P Eaves CJ. Cancer stem cells: an evolving concept. Nat Rev Cancer. (2012) 12:133–43. 10.1038/nrc3184 22237392

[31] Phillips TM McBride WH Pajonk F. The response of CD24(-/low)/CD44+ breast cancer-initiating cells to radiation. J Natl Cancer Inst. (2006) 98:1777–85. 10.1093/jnci/djj495 17179479

[32] Hatina J Schulz WA. Stem cells in the biology of normal urothelium and urothelial carcinoma. Neoplasma. (2012) 59:728–36. 10.4149/neo_2012_089 22862174

[33] Goodwin Jinesh G Willis DL Kamat AM. Bladder cancer stem cells: biological and therapeutic perspectives. Curr Stem Cell Res Ther. (2014) 9:89–101. 10.2174/1574888X08666131113123051 24236543

[34] Jinesh GG Choi W Shah JB Lee EK Willis DL Kamat AM. Blebbishields, the emergency program for cancer stem cells: sphere formation and tumorigenesis after apoptosis. Cell Death Differ. (2013) 20:382–95. 10.1038/cdd.2012.140 23175184 PMC3569985

[35] Moon RT Kohn AD De Ferrari GV Kaykas A. WNT and beta-catenin signalling: diseases and therapies. Nat Rev Genet. (2004) 5:691–701. 10.1038/nrg1427 15372092

[36] Vincan E Barker N. The upstream components of the Wnt signalling pathway in the dynamic EMT and MET associated with colorectal cancer progression. Clin Exp Metastasis. (2008) 25:657–63. 10.1007/s10585-008-9156-4 18350253

[37] Zhang Q Lou Y Zhang J Fu Q Wei T Sun X. Hypoxia-inducible factor-2α promotes tumor progression and has crosstalk with Wnt/β-catenin signaling in pancreatic cancer. Mol Cancer. (2017) 16:119. 10.1186/s12943-017-0689-5 28705232 PMC5512744

[38] Muñoz A Barceló JR López-Vivanco G. Chemotherapy for bladder cancer. N Engl J Med. (2003) 349:2272–3. 10.1056/NEJM200312043492323 14657442

[39] Takeishi S Nakayama KI To wake up cancer stem cells, or to let them sleep, that is the question. Cancer Sci. (2016) 107:875–81. 10.1111/cas.12958 27116333 PMC4946711

[40] Essers MA Trumpp A. Targeting leukemic stem cells by breaking their dormancy. Mol Oncol. (2010) 4:443–50. 10.1016/j.molonc.2010.06.001 20599449 PMC5527930

