# Supplementary material for: Activation of Glucocorticoid Receptor Inhibits the Stem-Like Properties of Bladder Cancer via Inactivating the β-Catenin Pathway
Source: Front Oncol. 2020 Aug 5;10:1332. doi: 10.3389/fonc.2020.01332 (PMC7419687; doi:10.3389/fonc.2020.01332)
Supplement: Supplementary file 5 [file Presentation_1.pdf]

Supplementary table captions

Supplementary table 1. The original data of reactive oxygen species assay.

Supplementary table 2. The original data for numbers of spheres.

Supplementary table 3. The original data of QPCR.

Supplementary table 4. The original data of the rates of CD44 positive cells in the flow cytometry analysis.
